# Hospital Note

**Published:** 1905-07

**Authors:** 


### HOSPITAL NOTE.

THE Buffalo State Hospital will in future be governed by a board of managers, as was formerly the case. In accordance with a law passed last winter, Governor Higgins has appointed the following named managers for this institution: Joseph P. Dudley, Dr. William C. Krauss, Nathan Wolf, George H. Kennedy, Mrs. Tracy C. Becker, and Miss Margaret F. Rochester, Buffalo, and John T. Darrizon, Lockport.

